# Prevention and Treatment of Intestinal Failure-Associated Liver Disease in Children

**DOI:** 10.3390/nu10060664

**Published:** 2018-05-24

**Authors:** Lorenzo Norsa, Emanuele Nicastro, Angelo Di Giorgio, Florence Lacaille, Lorenzo D’Antiga

**Affiliations:** 1 Pediatric Gastroenterology Hepatology and Nutrition, Ospedale Papa Giovanni XXIII, 24127 Bergamo, Italy; enicastro@asst-pg23.it (E.N.); adigiorgio@asst-pg23.it (A.D.G.); ldantiga@asst-pg23.it (L.D.); 2 Pediatric Gastroenterology Hepatology and Nutrition, Hôpital Necker Enfants Malades, 75015 Paris, France; florence.lacaille@aphp.fr

**Keywords:** intestinal failure, liver disease, parenteral nutrition, children, prevention, treatment

## Abstract

Intestinal failure-associated liver disease (IFALD) is a threatening complication for children on long-term parenteral nutrition because of intestinal failure. When progressive and intractable, it may jeopardize intestinal rehabilitation and lead to combined liver and intestinal transplantation. The institution of dedicated intestinal failure centers has dramatically decreased the incidence of such complication. IFALD may rapidly fade away if very early management aimed at preventing progression to end-stage liver disease is provided. In this review, we address the etiology and risk factors of IFALD in order to introduce pillars of prevention (nutritional management and catheter-related infections control). The latest evidence of therapeutic strategies, such as medical and surgical treatments, is also discussed.

## 1. Introduction

Intestinal failure-associated liver disease (IFALD) is a serious and potentially life-threatening complication for children suffering from intestinal failure. The aim of the authors is to produce a review on the current evidences regarding this condition. A Pubmed-based research review was conducted using the terms: “intestinal failure-associated liver disease (IFALD)” and “children”. The authors selected valuable papers for the production of an expert’s critical review providing an update on IFALD in children. 

## 2. Characteristics of Intestinal Failure-Associated Liver Disease

The advent of parenteral nutrition (PN) has dramatically improved the life expectancy of children with (IF), defined as the reduction of functional gut mass below the necessary amount to provide adequate nutrient and fluid requirements for the maintenance of metabolic functions in adults and to warrant normal body growth in children [1]. 

In addition, the overall advance in neonatal intensive care skills has led to a steep drop in mortality of infants with short bowel syndrome and IF of other causes [2]. Thus, the number of PN-dependent children has increased, and, consequently, morbidity has risen. 

IFALD is the most frequent complication of long-term PN in children with IF of diverse etiology. IFALD, in most cases, is a preventable and reversible liver disorder, but still a potential cause of end-stage liver disease and one of the major indications to intestinal or combined liver and small bowel transplantation [3]. For these reasons, all efforts should be undertaken to identify the disease early on, in a stage where medical interventions are able to improve or revert its extent. 

IFALD can be best defined as “hepatobiliary dysfunction as a consequence of medical and surgical management strategies for intestinal failure, which can variably progress to end-stage liver disease, or can be stabilized or reversed with promotion of intestinal adaptation” [4]. This wording stresses the multifactorial pathogenesis of this condition, in which liver injury is the result of several conditions coexisting in the clinical scenario of the intestinal impairment. IFALD does not refer to the self-recovering neonatal cholestasis that occurs in up to 2% of neonatal intensive care unit admitted newborns, but is associated with short-term PN and prematurity [5]. For this reason, the term IFALD has largely replaced the previously used and perhaps misleading definition “PN-associated liver disease/cholestasis” (PNALD, PNAC). 

### 2.1. Epidemiology

Few studies have reported the prevalence of IFALD among patients with IF from primary digestive and non-digestive conditions. Although earlier studies reported a prevalence around 40–60% [6,7], more recent data unanimously indicate a lower rate. In a recent retrospective observation with a 14-year follow-up of 251 children on home PN, IFALD had a prevalence of 20%, occurring more frequently in patients with a primary digestive disease compared with patients with intestinal failure due to immune-deficiency or other non-digestive diseases, and progressing to end-stage liver disease in 10% of cases [8]. A similar prevalence was observed, over a 4-year study period, in a cohort of hospitalized patients receiving long-term PN, in which IFALD was diagnosed in 22% of children, leading to end-stage liver disease in 4%. Younger and premature children, those affected by primary digestive disease, or needing non-transplant surgery, and those with longer PN administration had the highest risk of IFALD [9]. Importantly, when IFALD presents with symptoms of liver disease, mortality is high, between 23–40% [9,10]. In a cohort from Boston Children’s Hospital where liver biopsy was systematically performed, cirrhosis was present in about 18% of children with IFALD, as well as associated with a longer duration of parenteral support [11]. 

Nowadays, with enhanced awareness and a multidisciplinary approach to IF, incidence of IFALD seems to have reduced, the impact of sepsis is smaller, and progression to end-stage liver disease is generally limited to children that cannot be fed enterally or that present other organs failure [12].

### 2.2. Diagnostic Considerations

Diagnosis of IFALD is usually made on clinical grounds in children requiring long-term PN for IF, in the presence of biochemical evidence of cholestasis. Since, like in many other liver diseases, IF-associated liver injury progresses in the absence of symptoms, the suspect should exhibit even mild biochemical changes. Conjugated hyperbilirubinemia as little as 1.2 mg/dL could represent the only initial sign that, if persistent, should drive the first preemptive interventions [3,4]. Early IFALD has been defined in some studies in the presence of transaminases and/or gamma-glutamyltransferase (GGT) (>1.5 upper limit of normal) and hyperbilirubinemia below 3 mg/dL, while the combination of persistent hyperbilirubinemia >6 mg/dL and the prolongation of prothrombin time mark the highest risk for severe IFALD and progression to end-stage liver disease [9,13]. However, a mild isolated raise in transaminases and a moderate hyper-GGT (≤4 upper limit of normal) can be present in patients with IF without confirmed IFALD [13]. Obviously, the statements of these different studies should be weighted according to the definition of IFALD formulated in each center.

Radiology has little use in IFALD, and is usually limited to the assessment of splenomegaly in the advanced disease and to the study of the liver texture for fibrotic changes.

Liver biopsy is not usually needed for the diagnosis, and should be limited to selected cases for staging purposes or surgical decisions. Although the presence of cirrhosis on liver biopsy is not a determinant of survival, and children with cirrhosis can remain stable for long periods of time, the complications of cirrhosis and portal hypertension are relevant in subjects with IF [11]. 

When available, histology is characterized by cholestasis, features of biliary obstruction (portal inflammation, edema, ductular proliferation), and micro- and macrovescicular zone 1 steatosis. In advanced disease, fibrosis has a biliary pattern, beginning with portal expansion and progressing to periportal fibrosis, ultimately ending in porto-portal bridges [14].

## 3. Risk Factors for IFALD Development

**Figure 1:**
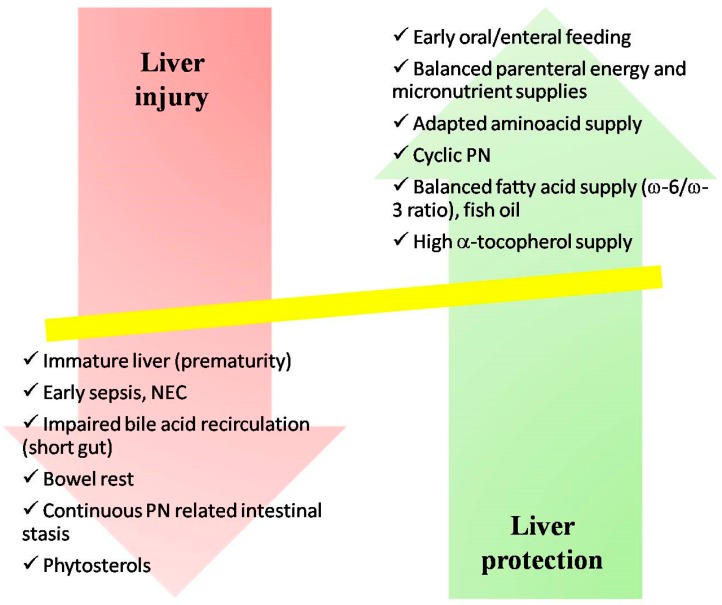
Factors affecting intestinal failure-associated liver disease. PN: parenteral nutrition, NEC: necrotizing enterocololitis.

The cause of IFALD is complex and poorly understood. Many risk factors have been implicated in the pathogenesis of IFALD but, interestingly, no single factor has been implicated as the main culprit, supporting the hypothesis of a multifactorial etiology [15] (Figure 1). 

Liver disease is more common in infants and neonates than in adults, suggesting that the neonatal liver may be more susceptible to injury. The risk factors for IFALD can be divided into two categories: patient-related and PN-related factors. Many studies have been performed to explain the role of these factors in the pathogenesis of IFALD, but none has been carefully studied in a prospective controlled clinical trial, therefore the aforementioned studies remain speculative [15].

### 3.1. Patient-Related Risk Factors

Patient-dependent risk factors include the degree of liver maturation (prematurity), early or recurrent sepsis, small intestinal bacterial overgrowth (SIBO), paucity of oral and enteral nutrition, and abdominal surgery with prolonged maintenance of stomas interrupting the entero-hepatic circulation [4].

Residual small bowel length after surgery has been described as a prognostic factor on PN weaning, thus exposing the patient to a higher risk of PN complications [16]. However, a recent study failed to demonstrate a correlation between residual small bowel and IFALD in children with very short bowel syndrome [17].

#### 3.1.1. Prematurity

The first report associating IF with cholestasis leading to cirrhosis in premature babies receiving PN was published four decades ago, and since then many further studies have followed [18].

Prematurity is an established risk factor for IFALD, with a prevalence of 25–33% of IF children [19]. Recent studies have suggested that being small for gestational age (SGA) is an independent risk factor for developing cholestasis; however, these results have not been confirmed by other authors and therefore remain controversial [20,21]. Premature babies have immature livers with incompletely expressed enzymatic activity, an inadequate bile salt uptake and excretion, and also an inadequate production of glutathione [22]. Another possible mechanism could be a cystathionase deficiency in preterm infants. This enzyme is involved in the conversion of methionine to cysteine and hence to taurine [23]. Studies on rabbit models have demonstrated that the lack of taurine and its precursors induces metabolic and structural liver damage [24].

Furthermore, the infantile liver is probably more susceptible to damage from lipid peroxidation and sepsis. Among premature babies, those who develop necrotizing enterocolitis (NEC) seem to be more susceptible to developing IFALD because they are exposed to further risk factors including high rate of sepsis, intestinal obstruction, short bowel, and disrupted enterohepatic bile acid circulation, high glucose and lipid intake to meet energy needs, and the requirement of continuous rather than cyclical PN infusion [4].

Premature babies can also develop a decreased portal vein flow, which could act directly on the liver, causing ischemia, or indirectly through remote organ damage caused by intestinal ischemia [25,26].

#### 3.1.2. Early and/or Recurrent Sepsis

Episodes of sepsis play a crucial role for the development of liver injury in children with IF [27].

Sepsis appears to be an independent risk factor for IFALD and each septic episode is associated with a 3.2-fold increase in the risk of developing jaundice [28,29].

These patients have a high rate of sepsis, which is related to multiple factors. First of all, children with PN-dependent intestinal failure require the placement and frequent handling of central venous catheters to permit the delivery of nutrients, and therefore they are susceptible to sepsis from catheter-related bloodstream infections (CRBSIs). Among the causing agents of CRBSIs, Gram-negative bacteria may increase liver dysfunction [30]. The incidence of CRBSIs among children on long-term PN ranges from 1.3 to 10.2 per 1000 catheter days, with a higher risk in children with short bowel syndrome (SBS) aged <1 year [31].

A multiple regression analysis showed that the duration of time the catheters were in place and the duration of PN administration were the most significant contributors to cholestasis [32]. Thus, the presence of a central venous access is a clear key risk factor for morbidity and mortality among patients with IF.

Other sources of bacterial sepsis in these children are feeding enterostomies and stomas that may result in bacterial translocation to the bloodstream [33].

Recurrent sepsis has been shown to be a risk factor for liver injury, in part because the endotoxins released during sepsis act directly or indirectly through the production of inflammatory cytokines on bile transport proteins, impairing biliary excretion and, as a consequence, leading to cholestasis [34]. In addition, murine models of intestinal damage have shown that lipopolysaccharides (LPS) located on the outer membrane of Gram-negative bacteria are associated with Toll-like receptor 4 (TLR-4) activation in Kupffer cells promoting apoptosis, cholestasis, and fibrosis [30].

CRBSIs rate is also crucial for the development of liver damage. A correlation between central catheter infection and liver fibrosis was demonstrated in a study on 30 children on prolonged PN programs. The patients were divided two groups based on the severity of the liver disease on liver biopsy (group A: severe fibrosis or cirrhosis; group B: mild fibrosis). Comparing the two groups, the incidence of infections was significantly higher in group A than in group B: 3.2 ± 0.3/study-year vs. 1.5 ± 0.2/study-year (*p* < 0.001). Among the microorganisms responsible for infections listed in this study, a majority were Staphylococcus species and Gram-negative bacilli [35].

#### 3.1.3. Small Intestinal Bacterial Overgrowth (SIBO)

Following significant bowel resection, the remaining bowel is prone to increasing its caliber due to strictures or ineffective propulsive waves. In this setting, the small bowel dilation along with poor intestinal motility and the inefficient absorption of nutrients promote SIBO. It has been observed in a large series of infants and children with IFALD that this bacterial overgrowth is related to the severity of small intestine inflammation, presumably impairing the intestinal barrier function and promoting the absorption of small molecules across the small bowel mucosa [36]. This relationship between bacterial overgrowth and inflammatory pathways was clearly demonstrated in a PN-dependent mouse model in which a shift in the intestinal microbiota was associated with intestinal epithelial cell apoptosis, increased expression of mucosal proinflammatory cytokines, and a loss of intestinal barrier function [37].

The authors also demonstrated that an overgrowth of Erysipelotrichaceae and Bacteroidetes species was observed in mouse models that developed cholestasis and liver injury, which was associated with increased intestinal permeability and the absorption of lipopolysaccharide. A combination of four oral antibiotics, resulting in the significant suppression of intestinal microbiota, prevented liver injury, cholestasis, and the activation of hepatic macrophages [30].

This confirms that an increased intestinal permeability is another important factor in the pathogenesis of IFALD because it promotes the bacterial translocation and bacteremia, or simply the absorption of bacterial cell wall products, capable of activating the innate immune system and favoring the episodes of sepsis which lead to liver injury.

The relationship between oral and enteral nutrition and SIBO is very weak. On one hand, a lack of intake could lead to intestinal dysmotility and, on the other hand, overfeeding can result in intestinal dilatation. Both of these are associated with a higher risk of developing SIBO [27].

#### 3.1.4. Paucity of Oral and Enteral Nutrition

Enteral nutrition (EN) is considered an important tool for the prevention or reversal of IFALD. It decreases the rate of the intestinal adaptation promoting the complex set of time- and segment-specific compensatory changes that maintain the bowel absorptive function (i.e., the increase in small intestinal mucosal thickness, villus length, crypt depth) [38], ultimately decreasing the requirements of PN. Moreover, EN does not favor bacterial overgrowth [32]. Unfortunately, patients with intestinal failure are commonly unable to tolerate substantial enteral nutrient stimulation. The lack of enteral feeding impairs the enterohepatic circulation and bile acid secretion/absorption, thus leading to mucosal atrophy, and increasing the risk of bacterial translocation. It has been demonstrated that hormones stimulated by EN (e.g., cholecystokinin, motilin, gastrin, glucose-dependent insulin tropic polypeptide, and glucagon) are decreased in patients on PN [22,32].

### 3.2. Parenteral Nutrition Related Risk Factors

Children with severe intestinal failure and prolonged dependence on PN are susceptible to the development of IFALD. In a study on 90 patients with permanent IF, the authors demonstrated that the factors strongly associated with the onset of chronic cholestasis were the duration of PN, a dose of lipids (triglycerides rich in long-chain ω-6) >1 g/kg/day, and a length of functional bowel <50 cm [39].

The main factors related to PN which may determine the development of liver injury in children with IF are:

- inappropriate use of lipid emulsions
- lack of antioxidants and the presence of phytosterols in the lipid emulsion
- micronutrient imbalances and the administration of excessive amounts of glucose
- duration of the infusion period

#### 3.2.1. Inappropriate Use of Lipid Emulsions

Several factors should be taken into consideration when choosing a fat emulsion for parenteral use, including the content in essential fatty acids (FAs), the ratio of ω-6/ω-3, the polyunsaturated fatty acid (PUFAs) content, the amount of medium-chain triglycerides (MCTs), and the quantity of α-tocopherol and phytosterols [27].

Over the past 20 years, considerable animal and human data have shown that the use of inappropriate lipid emulsions in PN, such as soybean oil-based emulsions (SOE), represents the major factor responsible for the development of liver injury in children with IF [27]. Different mechanisms have been proposed to explain how the lipid emulsions can cause liver injury in children on PN.

First, the role of phytosterols is discussed. SOE contain high amounts of phytosterols, or plant-based cholesterol-like compounds found in vegetable oils, which disrupt the bile acid homeostasis and contribute to the development of cholestasis [40]. Clinical studies in neonates and children with IFALD receiving PN demonstrated that serum stigmasterol (and other phytosterols) was markedly elevated as compared with infants and children on PN who did not develop IFALD. However, in these clinical studies it was difficult to determine whether the elevated phytosterols were the cause or the result of the cholestasis [41].

Secondly, the dose of lipids contained in PN is considered. Doses of intravenous (IV) SOE ≥1 g/kg/day have also been associated with increased risk of IFALD in mixed adult and pediatric home PN (HPN) cohorts [22]. Young children on PN require a greater dose of fat emulsion per kilogram of body weight to provide energy requirements, warrant normal neurological development, and prevent essential fatty acid deficiency. Besides, as discussed later, the lower amount of antioxidants contained in SOE compared to fish oil-based emulsion (FOE) may expose the liver to the risk of injury from oxidative stress and lipid peroxidation [15].

Third, the role of ω-6/ω-3 polyunsaturated fatty acids is noted. Both are essential polyunsaturated fatty acids (PUFAs) that must be derived from the diet and cannot be made by human and other mammals metabolism because of the lack of endogenous enzymes for omega-3 desaturation [27].

SOE are rich in ω-6 PUFAs and the eicosanoid products derived from ω-6 PUFAs (such as prostaglandin and leukotriene synthesized from arachidonic acid) are potent mediators of thrombosis and inflammation. In contrast, the ω-3 PUFAs found in fish oil (FO) products but not in plant oils (such as α-linolenic acid) are converted into anti-inflammatory derivatives. Thus, an unbalanced ω-6/ω-3 ratio in favor of ω-6 PUFAs is highly prothrombotic and proinflammatory, while a ratio in favor of ω-3 is protective from several diseases including atherosclerosis, obesity, diabetes, and hepatic cholestasis [42]. Recent studies showed that the infusion of fat emulsions based on pure FO containing ω-3 PUFAs (Omegaven^®^, Fresenius Kabi, Bad Homburg, Germany) allowed the reduction of the intake of proinflammatory ω-6 and phytosterols while increasing the amounts of a-tocopherol, a powerful antioxidant [27]. Recently, in a study performed on a culture of human hepatocyte cells, the authors demonstrated the anti-inflammatory and hepatoprotective effect of ω-3 PUFAs by abating inflammatory cytokine signaling such as TGF-β, IL-1, and TNF-α [43].

However, FO provides less essential ω-6 fatty acids than that presently recommended in infants and young children, therefore it should not be recommended as the unique source of lipids for a long period of time. The most proper lipid emulsion might be represented by a mixture of soybean oil (30%), coconut oil (30%), olive oil (25%), and fish oil (15%) (SMOF-lipid^®^, Fresenius Kabi, Bad Homburg, Germany), having a correct balance between ω-6/ω-3 polyunsaturated fatty acids. In fact, a reduced dose of SOE, the addition of FOE, and fat emulsions designed with a mixture of soy oil, medium-chain triglycerides, olive oil, and fish oil (SMOF) have been considered as potential therapies for children on home parenteral nutrition who develop IFALD [27].

#### 3.2.2. Lack of Antioxidants

Lack of antioxidants is another crucial factor which may favor the onset of hepatic cholestasis in children with IF. Vitamin E (tocopherol) is an important natural antioxidant contained in lipid emulsions. Its different forms are known as α, β, γ, and δ-tocopherol, according to the methyl or proton groups that are bound to their benzene rings. Among these forms, the most powerful and biologically active form is α-tocopherol (mainly contained in FOE), while γ-tocopherol (contained in soybean oil emulsions) has 25% of the antioxidant power as compared with α-tocopherol [44]. SMOF-lipid^®^ emulsion contains soybean oil (30%), coconut oil (30%), olive oil (25%), and fish oil (15%), with an important addition of a-tocopherol (200 mg/L), equal to that found in the emulsion based only on FO (Omegaven^®^) [27].

#### 3.2.3. Micronutrient Imbalances

All PN components may potentially cause liver injury in children on long-term PN. It is known that an excess of energy (delivered as either dextrose or lipids) may cause hepatic steatosis. At a high infusion rate, raised plasma glucose concentrations result in increased plasma insulin which, as a consequence, stimulates the hepatic lipogenesis (fatty acid synthesis, glycogen synthesis, and fatty acid esterification) while depressing fatty acid oxidation, glycogenolysis, and gluconeogenesis [45]. Fat supplied intravenously is carried by liposomes, rather than chylomicrons, with an “artificial” delivery of fat to the liver. Thus, fat infusion results in steatosis, even when given in physiological amounts, and even more so when given in excess. This fat is typically seen in Kupffer cells and hepatic lysosomes [46]. Higher dextrose intake also increased the prevalence of cholestasis [47].

An imbalanced infusion of amino acids may be associated with the development of IFALD but the mechanism by which the amino acids may cause liver damage is not well understood. Elevated serum methionine levels, without a corresponding increase in its metabolites, was associated with cholestasis in a rabbit model, while the deficiency of taurine and cysteine may also play a role, especially in premature infants [48,49].

#### 3.2.4. Duration of the Infusion Period and PN Cycling

Continuous infusion of PN over a 24-h period represents a risk factor for IFALD, while the cycling of PN, which involves the provision of a total daily PN volume in less than 24 h, is a protective factor. Cycling PN is recommended for patients who are expected to be nourished with prolonged courses of PN (>30 days) and whose cardiac, renal, and endocrine function can tolerate shifts in fluid and dextrose infusion rates [50].

Previous studies showed some advantages of a cyclic infusion compared to a continuous infusion, including higher lipid oxidation, lower dextrose use, the reduction of serum liver enzymes and conjugated bilirubin, and the reduction in both hyperinsulinemia and fat deposition in the liver. Cycling should be adjusted as enteral intake increases, as well as tailored to patient age, because neonates often have a diminished glycemic reserve. Although cyclic PN was shown to be well tolerated even in young children, in newborns and infants the risk of hypoglycemia may limit its application [4]. 

## 4. Prevention of IFALD

The prevention of IFALD starts from the early management of the aforementioned risks factors. This should be started from the day of intestinal failure diagnosis, involving a multidisciplinary team composed of a neonatologist, pediatric gastroenterologist, surgeons, specialized PN nurses, and dietitians. Specialized intestinal rehabilitation programs have dramatically reduced the incidence of PN complications [16].

### 4.1. Nutritional Management

Animal models have demonstrated that oral feeding promotes epidermal growth factor (EGF) release from salivary glands and increases the gastrointestinal secretion of trophic factors, thus promoting small intestine adaptation [51,52].

These evidences suggested an important role of very early oral feeding stimulation in order to prevent intestinal failure complications, particularly IFALD [53]. Another important reason to encourage the early oral feeding in these children is to avoid food aversion that in the long-term may jeopardize the success of IF resolution and weaning off PN.

The first evidences of a protective role of early enteral feeding on parenteral nutrition-related liver damage were published in the late 80s in two randomized controlled trials (RCT) that enrolled very low birth weight infants [54,55]. Their findings, in the early 90s, led to the creation of an animal model that confirmed that enteral supplementation was protective against liver damage induced by parenteral nutrition [56]. Later on, a retrospective cohort study suggested that that early feeding after surgery could impact parenteral nutrition weaning, minimizing the risk of IFALD development [19]. These evidences supported the recommendation of early enteral feeding in children soon after surgery [4].

Very recently, an American team confirmed that greater initial enteral nutrition volumes and daily feeding advancement, if tolerated, decreased the odds ratio of developing IFALD in children [57]. 

A specifically designed retrospective study was able to confirm the superiority of breast-milk in preventing IFALD [58], making it the best nutritional option for children with intestinal failure. When breastfeeding was not possible, no evidence was produced in literature on the superiority of semi-elemental or elemental diet over polymeric formulas in achieving enteral autonomy [59].

Although there is only a very low level of evidence supporting this belief [60], continuous enteral feeding seems to be more tolerable in the early stages for children with intestinal failure. Previous studies have suggested that it is advisable to move to intermittent feeds administration only once half of the energy requirements are tolerated by continuous enteral feeding [4].

### 4.2. Central Venous Line Management

An important recommendation from the European Society of Pediatric Gastroenterology Hepatology and Nutrition is that each intestinal rehabilitation center must have a personalized strategy in order to prevent or to recognize and treat CRBSIs early on [4].

From the beginning of the current century, different teams have made great efforts to find a strategy to prevent CRBSIs.

The first step in order to minimize CRBSIs is the implementation of very strict education on the disinfection of hospital personnel and parents or caregivers while handling the central line [61].

Ethanol locks in central venous catheters were than tested in a meta analysis of four high-quality studies, and they were demonstrated to provide an 81% risk reduction of CRBSIs in IF children [62]. 

Lastly, two retrospective studies in children showed the efficacy of Taurolidine locks in preventing CRBSIs [63,64]. However results from randomized controlled trials in adults seem to indicate the superiority of a mixed taurolodine-citrate-heparin lock over a simple taurolodine lock to prevent CRBSIs [65,66].

### 4.3. Parenteral Nutrition Management

As previously mentioned, all components of parenteral nutrition could be implicated in the development of IFALD; thus, parenteral nutrition’s prescription plays a key role in IFALD prevention [4].

A specific study on resting energy expenditure in children with IFALD demonstrated that 80% of patients present hypo- and hypermetabolism if compared to the standard for sex, age, and weight [67].

These evidences suggest the importance of the close monitoring of energy requirements throughout the pediatric age, assessing growth parameters and providing prompt adaptation of PN energy intakes. 

Energy from carbohydrates should be balanced, since animal models and clinical experiences have demonstrated that increased carbohydrate-mediated energy could expose patients to hyperinsulinism and the risk of hepatic steatosis [68,69]. Nevertheless, a word of caution is necessary, because a recent study showed that high-carbohydrate and low-fat parenteral nutrition could reverse IFALD in children [70].

A very old study investigated the effect of protein intake on cholestasis in neonates on parenteral nutrition, and demonstrated that a higher intake of protein exposes patients to an earlier risk of developing cholestatic jaundice [71]. However, the dose of protein challenged in that study (3.6 g/kg) was far above the protein intake recommended for long-term parenteral nutrition in children with IF (1.5–2.5 g/kg) [72].

As previously mentioned, the lipidic component is the most important consideration in the prevention and treatment of IFALD.

Commercialized lipid emulsions are summarized in Table 1.

A metanalysis published in 2015 on this topic selected seven high-quality studies and concluded that FOE seem to have an effect in the treatment but not in the prevention of IFALD [73]. The growing interest of the scientific community on lipid emulsions in PN led the nutrition committee of the European Society of Pediatric Gastroenterology Hepatology and Nutrition to publish a position paper on this issue [74]. The conclusion of this paper was that the use of multicomponent, FOE may contribute to a decrease in total bilirubin levels in children with IF on prolonged PN, and that a pure FO supply combined with a decrease or interruption of soybean oil-based lipid emulsion may contribute to cholestasis recovery in children with PNALD [74].

Pure FOE (Omegaven^®^) do not contain the recommended amount of essential fatty acids and may not provide enough calories for growth. In Europe, the preferred lipid emulsions are currently a mixture of fish, olive, coconut, and soybean oil (SMOFlipid^®^) [22,75]. Even though the guidelines of the American society of parenteral and enteral nutrition suggest that more evidences are required in order to recommend it [22], SMOFlipids have been adopted by an increasing number of intestinal rehabilitation units in the United States. Fibrosis may, however, develop or worsen, despite a normal bilirubin level, as was seen in a small series of children on long-term PN, with some of them ultimately needing transplantation [76,77]. Furthermore, liver fibrosis can persist despite the normalization of biochemistry and PN weaning [78].

Total lipid content has also been suggested to be linked to IFALD development. Severe cholestasis has been linked to high doses (>2 g/kg/day), and reversed with the temporary suspension of fat [76], suggesting a role of lipid minimization in the treatment of IFALD [79,80]. This should be carefully balanced with the risk of inadequate energy supply and the risk of essential fatty acids deficiency [81].

Evidence of an effect of cyclic PN (meaning its interruption for at least few hours during the day) on liver function in patients with IF was first described in adults in a randomized controlled trial [82]. Even if this role was only confirmed in newborns [83,84,85], cyclic PN appears to be the preferred mode of administration for children with IF for all of the aforementioned positive metabolic effects [86].

**Figure 2:**
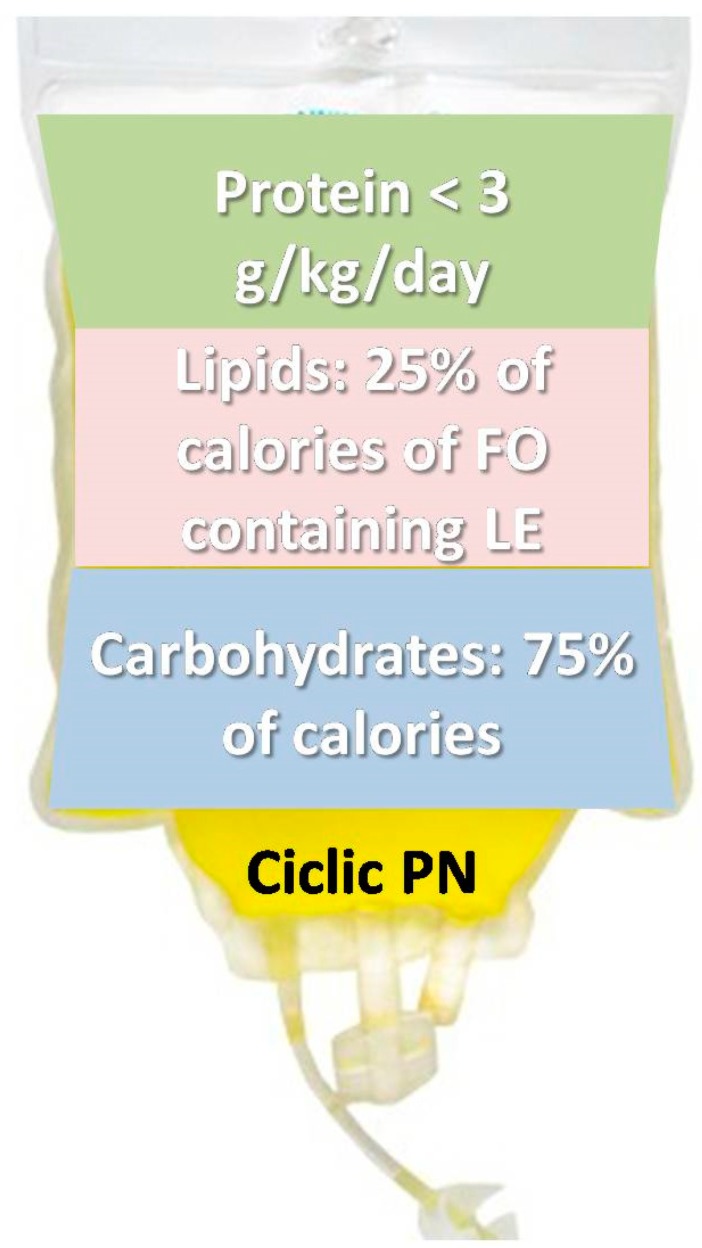
Suggestion of long-term parenteral nutrition preparation for children above 6 months of age. FO: fish oil, LE: lipid emulsion, PN: parenteral nutrition.

To summarize, a cyclic PN with an adequate caloric intake divided into 75% of non-protein calories as carbohydrates and 25% as fat through a composite fish oil-enriched lipid emulsion with >3 g/kg proteins seems to be the best option in order to prevent IFALD (Figure 2).

## 5. Treatment of IFALD

The treatment of IFALD could be divided into two phases. The first phase involves medical and surgical management, which aims to revert IFALD or increase intestinal absorption in order to promote PN weaning. The second is the ultimate treatment of IFALD, which could require liver transplantation (LTx) alone or combined with intestinal transplantation (ITx).

### 5.1. Medical Options

Only a few medical treatments have been available for IFALD, and clinical trials have shown little benefit from different medications. Ursodeoxycholic acid (UDCA) is traditionally used in cholestatic liver disease to stimulate bile flow (choleresis) [87]. The American guidelines suggest that further research is needed to strengthen the recommendation of UDCA administration in IFALD [22]. Nevertheless, UDCA is commonly prescribed in children with IFALD at a dose of 15–20 mg/kg/day. Evidence for the protective role of glutamine supplementation in very low birth weight infants are contradictory [88,89]. No evidence has been produced for children on long-term PN. Also, a study on the use of cholecystokinin [90] failed to demonstrate its efficacy in improving gallbladder stasis in neonates on parenteral nutrition.

An alternative medical strategy is the use of trophic factors such as growth hormone, insulin, and recently glucagon-like peptide 2 (GLP-2) to promote villous growth and thus decrease the need for PN and its side effects. Despite promising results in adults, studies on children did not demonstrate any clear advantage of the use of growth hormone [27] or oral insulin [91] in children with short bowel syndrome. On the other hand, the promising results of GLP-2 seen in adults with IF [92] seem to be confirmed by the first results produced in children [93]. Furthermore, very recently, GLP-2 was suggested to have a direct effect on IFALD throughout the alteration of bile acid metabolism [94]. 

### 5.2. Non-Transplant Surgery

The presence of the ileo-cecal valve (ICV) and colon influences the achievement of PN weaning, which in turn is considered the key to minimizing and in some cases even treating IFALD [95]. Further indirect evidences on this issue have suggested that IFALD may be prevented or improved by the early closure of a stoma when primary anastomosis is not feasible [16].

Small bowel lengthening procedure has been proposed for children with short bowel syndrome associated with bowel dilation. Serial Transverse Enteroplasty (STEP) and Longitudinal Intestinal Lengthening and Tailoring (LILT) have both been associated with improvement in liver function in a small series of patients [96,97]; however, in this setting it is always difficult to establish whether IFALD would eventually resolve irrespective of non-transplant surgery, since so far no controlled trials have been accomplished. However, both procedures should be very cautiously performed in children with advanced IFALD, due to portal hypertension as well as the risk of bleeding and surgical failure.

### 5.3. Transplantation

If in some cases home PN provides a better outcome in patients with IF, progressive IFALD still remains a clear indication to combined liver and intestinal transplantation which should not be underused or delayed [98]. Liver disease determines a shift from a condition of intestinal failure to proper nutritional failure [27]. 

However the debate is open on the evolution of indications for transplantation and graft selection [99].

It must be emphasized that in 2017 the long-term survival and quality of life after transplantation was still unsatisfactory [17,100]. In 2015, the Intestinal Transplant Registry reported the results of 1611 children (822 alive at last report), 1029 with a liver-containing graft. This cohort of children had 1- and 5-year patient survival rates of respectively 80% and 70%, and a 10-year graft survival of 55% [101]. The 10-year survival in large pediatric IF units is close to 100% in children with primary digestive diseases on long-term PN [8,102]. 

Graft selection highly depends from the degree of IFALD. As reported previously, staging IFALD severity could be very hard. Even if liver histology could be misleading [103], it is actually performed in most of the centers in the assessment before intestinal transplantation. The presence of portal hypertension, severe progressive cholestasis, and decompensated cirrhosis seem to be clear indications for a liver-containing graft [99]. 

If IFALD is milder and expressed by stable hyperbilirubinemia without signs of portal hypertension or progressive liver failure, isolated small bowel should be considered [104]. In addition, this option could lead to IFALD resolution thanks to PN weaning after transplantation. 

Isolated liver transplantation can be discussed as a life-saving procedure in a patient with end-stage IFALD, and reasonable expectations can be raised for intestinal autonomy after the resolution of portal hypertension. This could provide a shorter waiting list, lower immunosuppression after transplant, and better long-term outcomes [105]. Predictors of success of such a procedure were identified to be enteral nutrition tolerance >50% of total calories, small bowel lengths >25 cm, and age <2 years [106]. 

## 6. Conclusions

In conclusion, IFALD is a severe complication of intestinal failure that occurs mainly in the first few months of life and has a severe outcome. Preventive measures adopted in the last years, both on the side of the patient care and parenteral nutrition preparations, have led to a steep drop in its prevalence in this setting. A multidisciplinary approach including appropriate medical, surgical, nutritional, and nursing management could prevent and eventually revert the development of liver disease, which severely affects the outcomes of children with intestinal failure. 

## Figures and Tables

**Table 1:** Composition of fat emulsions for parenteral nutrition.

	Intralipid^®^	Medialipid^®^	ClinOleic^®^	SMOFlipid^®^	Omegaven^®^
Soybean oil %	100	50	20	30	0
MCT %	0	50	0	30	0
Olive oil %	0	0	80	25	0
Fish oil %	0	0	0	15	100
Phytosterols mg/L	350	200	330	48	0
α-tocopherol mg/L	38	<30	200	200	150–300
ω-3 fatty acids	+	±	+	++	+++
ω-6 fatty acids	+++	++	+	++	+

MCT: medium-chain triglycerides.
